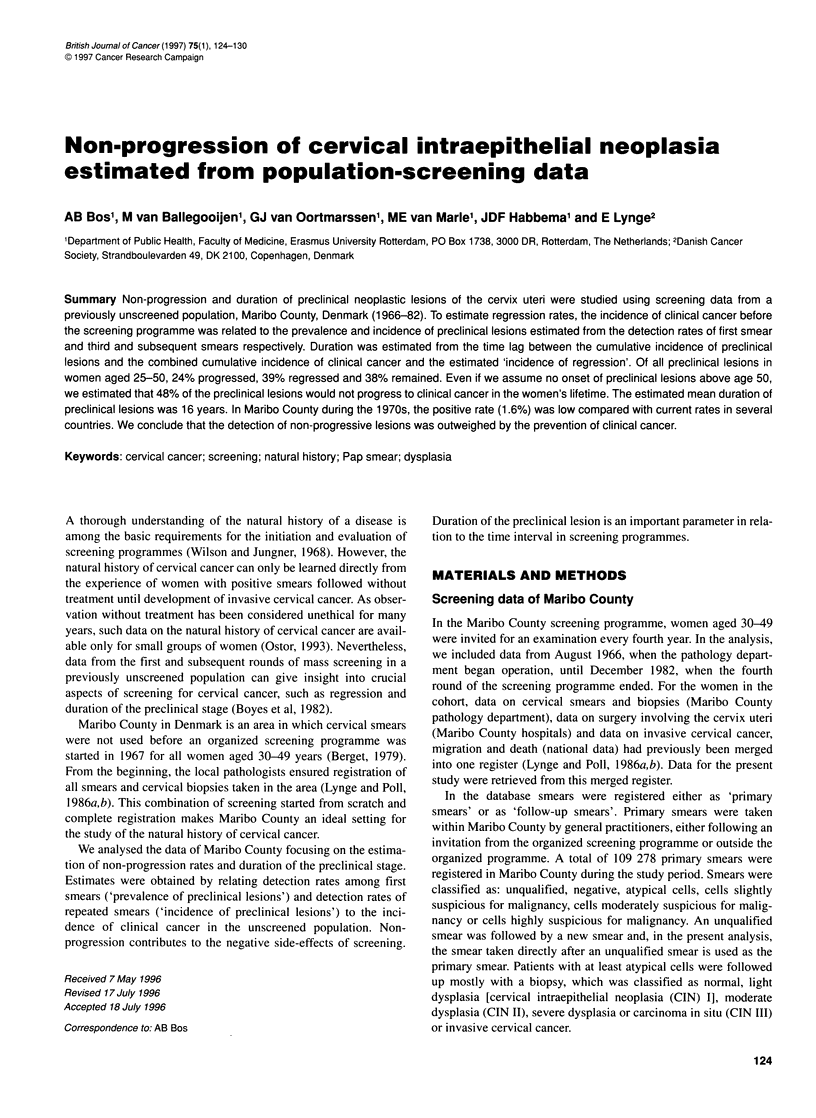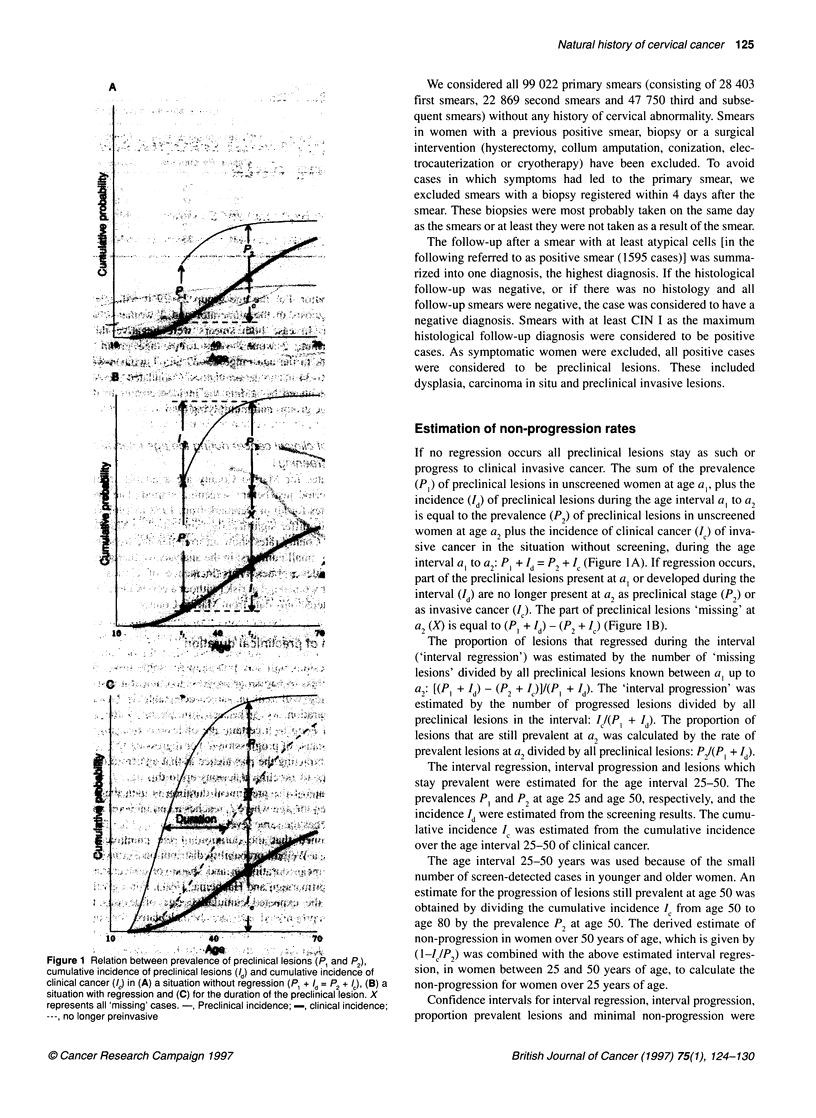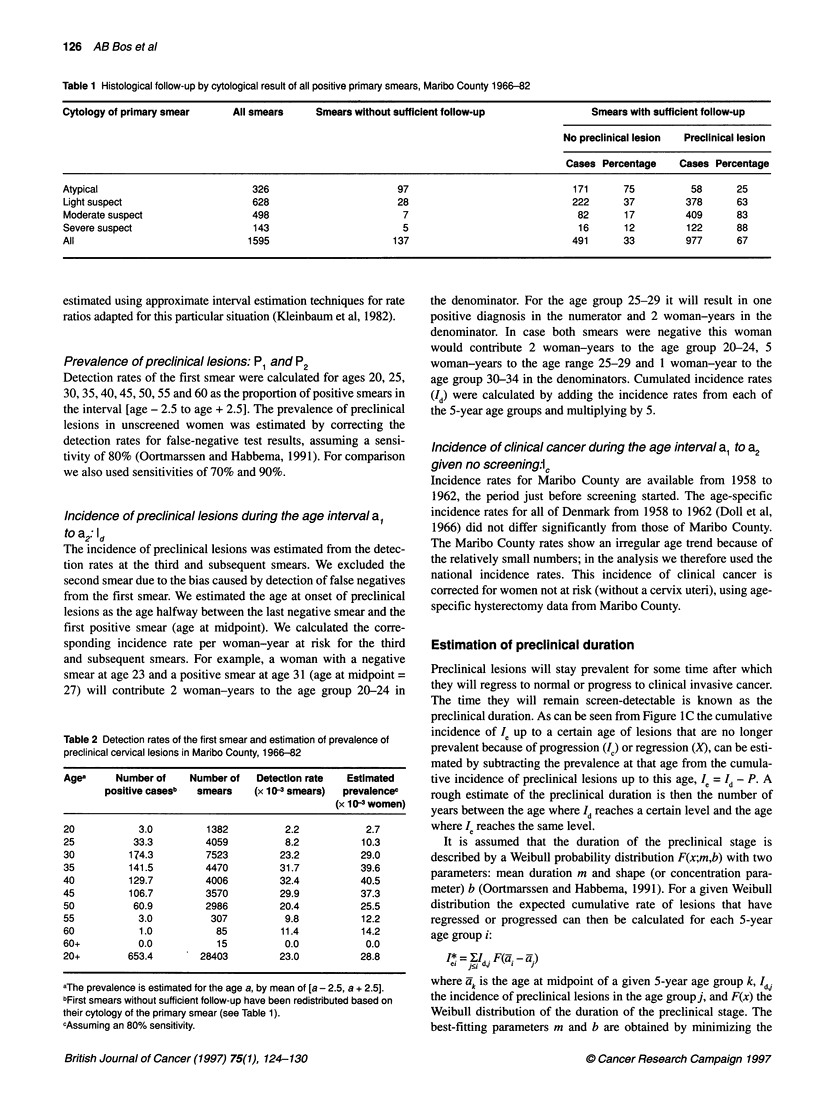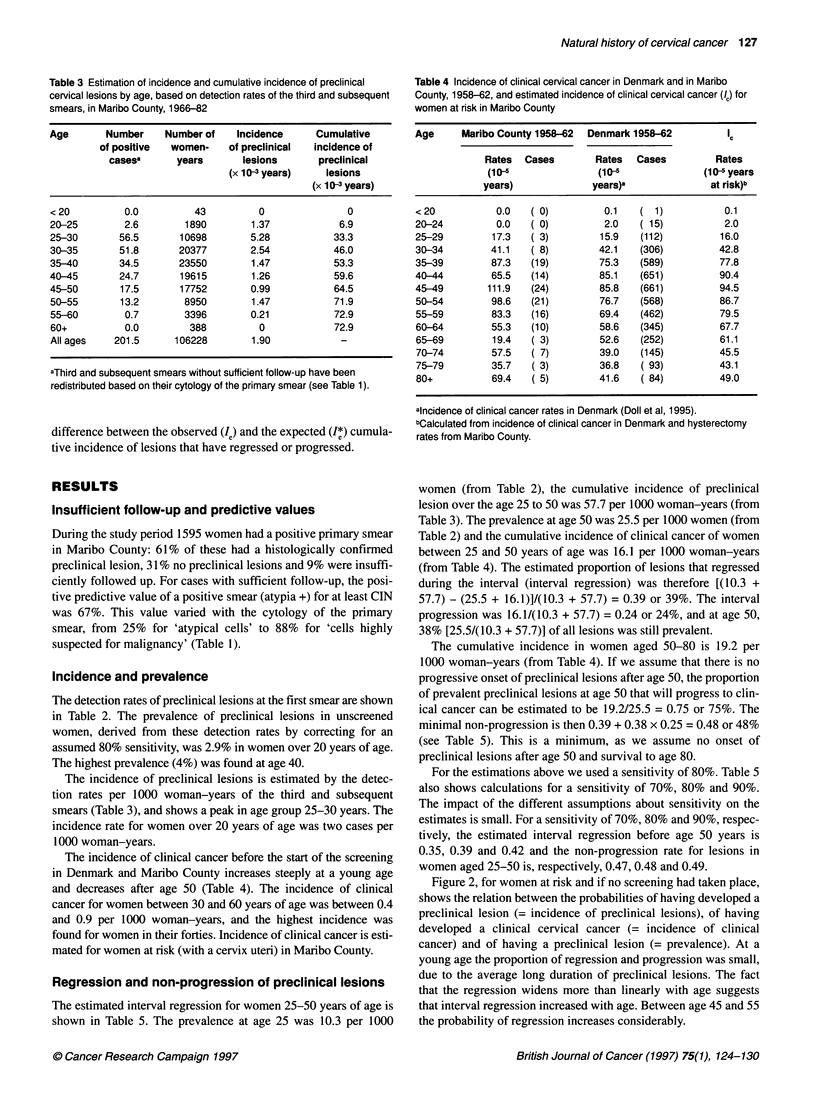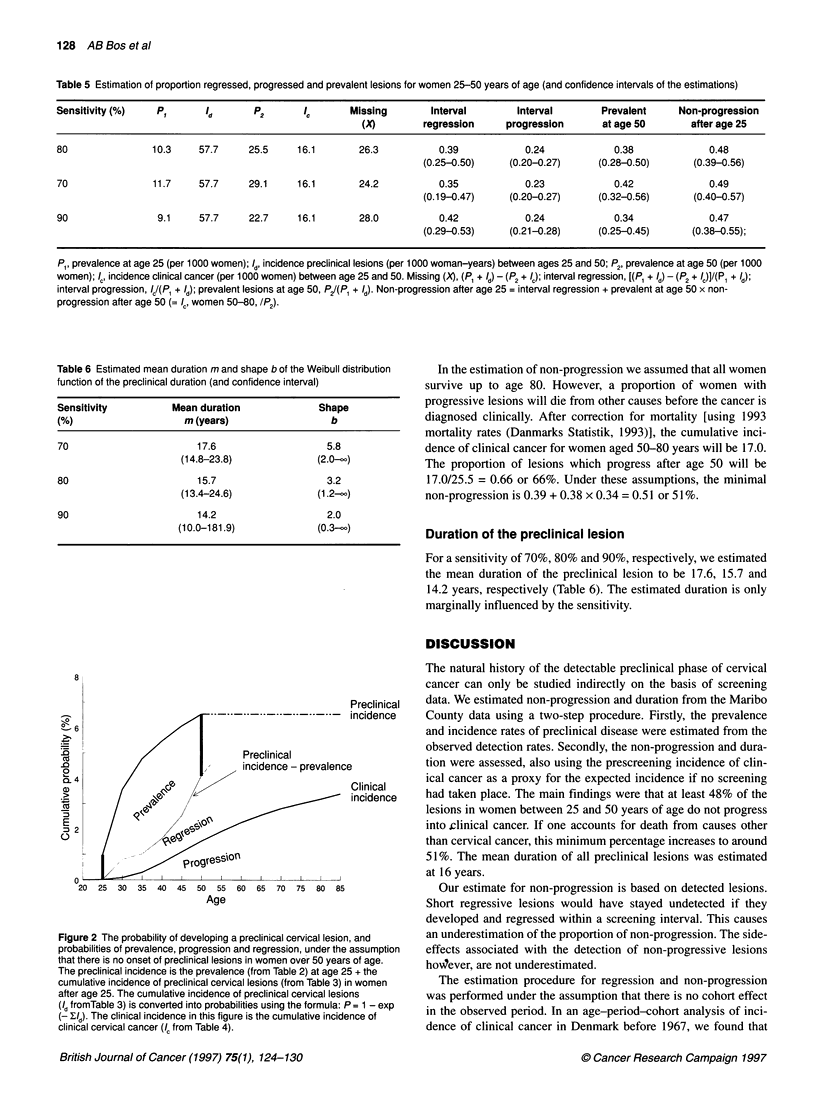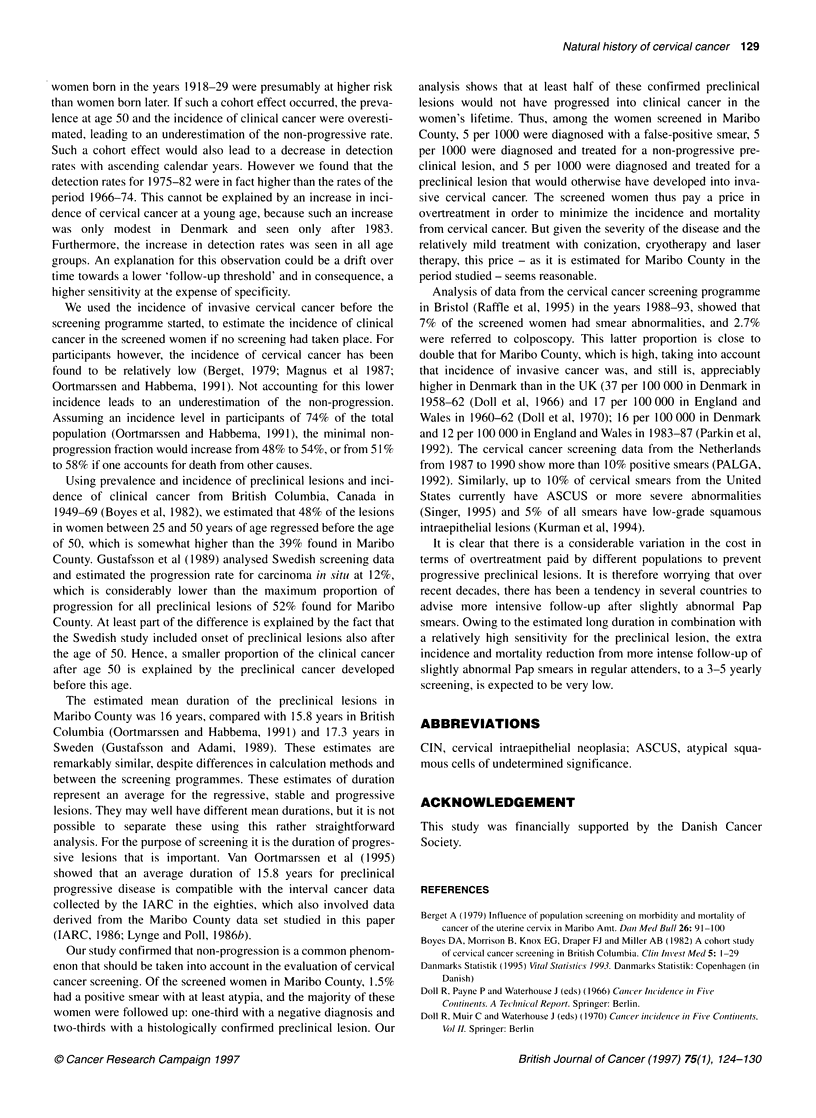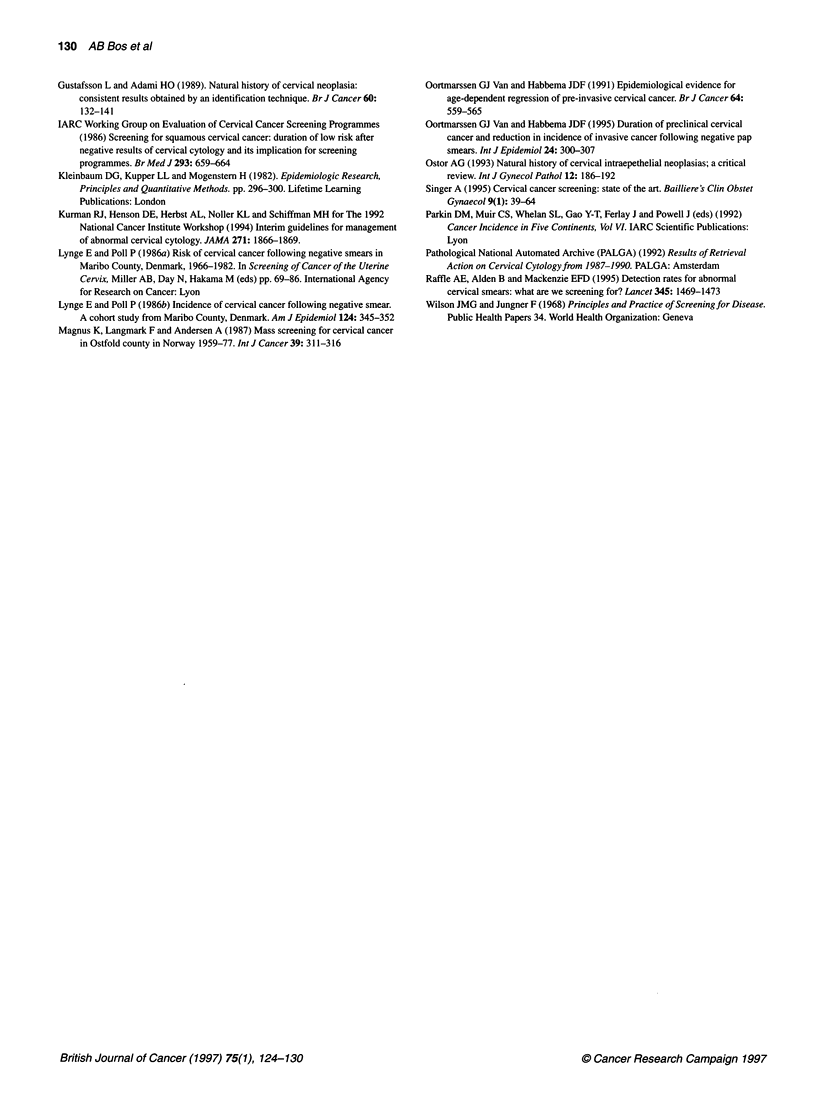# Non-progression of cervical intraepithelial neoplasia estimated from population-screening data.

**DOI:** 10.1038/bjc.1997.20

**Published:** 1997

**Authors:** A. B. Bos, M. van Ballegooijen, G. J. van Oortmarssen, M. E. van Marle, J. D. Habbema, E. Lynge

**Affiliations:** Department of Public Health, Faculty of Medicine, Erasmus University Rotterdam, The Netherlands.

## Abstract

**Images:**


British Journal of Cancer (1997) 75(1), 124–130
© 1997 Cancer Research Campaign

### Non-progression of cervical intraepithelial neoplasia estimated from population-screening data

**AB Bos[1], M van Ballegooijen[1], GJ van Oortmarssen[1], ME van Marle[1], JDF Habbema[1] and E Lynge[2]**

[1]Department of Public Health, Faculty of Medicine, Erasmus University Rotterdam, PO Box 1738, 3000 DR, Rotterdam, The Netherlands; [2]Danish Cancer Society, Strandboulevarden 49, DK 2100, Copenhagen, Denmark

**Summary** Non-progression and duration of preclinical neoplastic lesions of the cervix uteri were studied using screening data from a previously unscreened population, Maribo County, Denmark (1966–82). To estimate regression rates, the incidence of clinical cancer before the screening programme was related to the prevalence and incidence of preclinical lesions estimated from the detection rates of first smear and third and subsequent smears respectively. Duration was estimated from the time lag between the cumulative incidence of preclinical lesions and the combined cumulative incidence of clinical cancer and the estimated 'incidence of regression'. Of all preclinical lesions in women aged 25–50, 24% progressed, 39% regressed and 38% remained. Even if we assume no onset of preclinical lesions above age 50, we estimated that 48% of the preclinical lesions would not progress to clinical cancer in the women's lifetime. The estimated mean duration of preclinical lesions was 16 years. In Maribo County during the 1970s, the positive rate (1.6%) was low compared with current rates in several countries. We conclude that the detection of non-progressive lesions was outweighed by the prevention of clinical cancer.

**Keywords**: cervical cancer; screening; natural history; Pap smear; dysplasia

A thorough understanding of the natural history of a disease is among the basic requirements for the initiation and evaluation of screening programmes (Wilson and Jungner, 1968). However, the natural history of cervical cancer can only be learned directly from the experience of women with positive smears followed without treatment until development of invasive cervical cancer. As observation without treatment has been considered unethical for many years, such data on the natural history of cervical cancer are available only for small groups of women (Ostor, 1993). Nevertheless, data from the first and subsequent rounds of mass screening in a previously unscreened population can give insight into crucial aspects of screening for cervical cancer, such as regression and duration of the preclinical stage (Boyes et al, 1982).

Maribo County in Denmark is an area in which cervical smears were not used before an organized screening programme was started in 1967 for all women aged 30–49 years (Berget, 1979). From the beginning, the local pathologists ensured registration of all smears and cervical biopsies taken in the area (Lynge and Poll, 1986*a,b*). This combination of screening started from scratch and complete registration makes Maribo County an ideal setting for the study of the natural history of cervical cancer.

We analysed the data of Maribo County focusing on the estimation of non-progression rates and duration of the preclinical stage. Estimates were obtained by relating detection rates among first smears ('prevalence of preclinical lesions') and detection rates of repeated smears ('incidence of preclinical lesions') to the incidence of clinical cancer in the unscreened population. Non-progression contributes to the negative side-effects of screening. Duration of the preclinical lesion is an important parameter in relation to the time interval in screening programmes.

*Received 7 May 1996*
*Revised 17 July 1996*
*Accepted 18 July 1996*

*Correspondence to:* AB Bos

### MATERIALS AND METHODS

### Screening data of Maribo County

In the Maribo County screening programme, women aged 30–49 were invited for an examination every fourth year. In the analysis, we included data from August 1966, when the pathology department began operation, until December 1982, when the fourth round of the screening programme ended. For the women in the cohort, data on cervical smears and biopsies (Maribo County pathology department), data on surgery involving the cervix uteri (Maribo County hospitals) and data on invasive cervical cancer, migration and death (national data) had previously been merged into one register (Lynge and Poll, 1986*a,b*). Data for the present study were retrieved from this merged register.

In the database smears were registered either as 'primary smears' or as 'follow-up smears'. Primary smears were taken within Maribo County by general practitioners, either following an invitation from the organized screening programme or outside the organized programme. A total of 109 278 primary smears were registered in Maribo County during the study period. Smears were classified as: unqualified, negative, atypical cells, cells slightly suspicious for malignancy, cells moderately suspicious for malignancy or cells highly suspicious for malignancy. An unqualified smear was followed by a new smear and, in the present analysis, the smear taken directly after an unqualified smear is used as the primary smear. Patients with at least atypical cells were followed up mostly with a biopsy, which was classified as normal, light dysplasia [cervical intraepithelial neoplasia (CIN) I], moderate dysplasia (CIN II), severe dysplasia or carcinoma in situ (CIN III) or invasive cervical cancer.

**Figure 1** Relation between prevalence of preclinical lesions ($P_1$ and $P_2$), cumulative incidence of preclinical lesions ($I_d$) and cumulative incidence of clinical cancer ($I_c$) in (**A**) a situation without regression ($P_1 + I_d = P_2 + I_c$), (**B**) a situation with regression and (**C**) for the duration of the preclinical lesion. *X* represents all 'missing' cases. —, Preclinical incidence; ▬, clinical incidence; ---, no longer preinvasive

We considered all 99 022 primary smears (consisting of 28 403 first smears, 22 869 second smears and 47 750 third and subsequent smears) without any history of cervical abnormality. Smears in women with a previous positive smear, biopsy or a surgical intervention (hysterectomy, collum amputation, conization, electrocauterization or cryotherapy) have been excluded. To avoid cases in which symptoms had led to the primary smear, we excluded smears with a biopsy registered within 4 days after the smear. These biopsies were most probably taken on the same day as the smears or at least they were not taken as a result of the smear.

The follow-up after a smear with at least atypical cells [in the following referred to as positive smear (1595 cases)] was summarized into one diagnosis, the highest diagnosis. If the histological follow-up was negative, or if there was no histology and all follow-up smears were negative, the case was considered to have a negative diagnosis. Smears with at least CIN I as the maximum histological follow-up diagnosis were considered to be positive cases. As symptomatic women were excluded, all positive cases were considered to be preclinical lesions. These included dysplasia, carcinoma in situ and preclinical invasive lesions.

### Estimation of non-progression rates

If no regression occurs all preclinical lesions stay as such or progress to clinical invasive cancer. The sum of the prevalence ($P_1$) of preclinical lesions in unscreened women at age $a_1$, plus the incidence ($I_d$) of preclinical lesions during the age interval $a_1$ to $a_2$ is equal to the prevalence ($P_2$) of preclinical lesions in unscreened women at age $a_2$ plus the incidence of clinical cancer ($I_c$) of invasive cancer in the situation without screening, during the age interval $a_1$ to $a_2$: $P_1 + I_d = P_2 + I_c$ (Figure 1A). If regression occurs, part of the preclinical lesions present at $a_1$ or developed during the interval ($I_d$) are no longer present at $a_2$ as preclinical stage ($P_2$) or as invasive cancer ($I_c$). The part of preclinical lesions 'missing' at $a_2$ ($X$) is equal to $(P_1 + I_d) - (P_2 + I_c)$ (Figure 1B).

The proportion of lesions that regressed during the interval ('interval regression') was estimated by the number of 'missing lesions' divided by all preclinical lesions known between $a_1$ up to $a_2$: $[(P_1 + I_d) - (P_2 + I_c)]/(P_1 + I_d)$. The 'interval progression' was estimated by the number of progressed lesions divided by all preclinical lesions in the interval: $I_c/(P_1 + I_d)$. The proportion of lesions that are still prevalent at $a_2$ was calculated by the rate of prevalent lesions at $a_2$ divided by all preclinical lesions: $P_2/(P_1 + I_d)$.

The interval regression, interval progression and lesions which stay prevalent were estimated for the age interval 25–50. The prevalences $P_1$ and $P_2$ at age 25 and age 50, respectively, and the incidence $I_d$ were estimated from the screening results. The cumulative incidence $I_c$ was estimated from the cumulative incidence over the age interval 25–50 of clinical cancer.

The age interval 25–50 years was used because of the small number of screen-detected cases in younger and older women. An estimate for the progression of lesions still prevalent at age 50 was obtained by dividing the cumulative incidence $I_c$ from age 50 to age 80 by the prevalence $P_2$ at age 50. The derived estimate of non-progression in women over 50 years of age, which is given by $(1-I_c/P_2)$ was combined with the above estimated interval regression, in women between 25 and 50 years of age, to calculate the non-progression for women over 25 years of age.

Confidence intervals for interval regression, interval progression, proportion prevalent lesions and minimal non-progression were

© Cancer Research Campaign 1997

**Table 1** Histological follow-up by cytological result of all positive primary smears, Maribo County 1966–82

| Cytology of primary smear | All smears | Smears without sufficient follow-up | Smears with sufficient follow-up | | | |
|---|---|---|---|---|---|---|
| | | | No preclinical lesion | | Preclinical lesion | |
| | | | Cases | Percentage | Cases | Percentage |
| Atypical | 326 | 97 | 171 | 75 | 58 | 25 |
| Light suspect | 628 | 28 | 222 | 37 | 378 | 63 |
| Moderate suspect | 498 | 7 | 82 | 17 | 409 | 83 |
| Severe suspect | 143 | 5 | 16 | 12 | 122 | 88 |
| All | 1595 | 137 | 491 | 33 | 977 | 67 |

estimated using approximate interval estimation techniques for rate ratios adapted for this particular situation (Kleinbaum et al, 1982).

### *Prevalence of preclinical lesions:* $P_1$ *and* $P_2$

Detection rates of the first smear were calculated for ages 20, 25, 30, 35, 40, 45, 50, 55 and 60 as the proportion of positive smears in the interval [age – 2.5 to age + 2.5]. The prevalence of preclinical lesions in unscreened women was estimated by correcting the detection rates for false-negative test results, assuming a sensitivity of 80% (Oortmarssen and Habbema, 1991). For comparison we also used sensitivities of 70% and 90%.

### *Incidence of preclinical lesions during the age interval* $a_1$ *to* $a_2$*:* $I_d$

The incidence of preclinical lesions was estimated from the detection rates at the third and subsequent smears. We excluded the second smear due to the bias caused by detection of false negatives from the first smear. We estimated the age at onset of preclinical lesions as the age halfway between the last negative smear and the first positive smear (age at midpoint). We calculated the corresponding incidence rate per woman–year at risk for the third and subsequent smears. For example, a woman with a negative smear at age 23 and a positive smear at age 31 (age at midpoint = 27) will contribute 2 woman–years to the age group 20–24 in the denominator. For the age group 25–29 it will result in one positive diagnosis in the numerator and 2 woman–years in the denominator. In case both smears were negative this woman would contribute 2 woman–years to the age group 20–24, 5 woman–years to the age range 25–29 and 1 woman–year to the age group 30–34 in the denominators. Cumulated incidence rates ($I_d$) were calculated by adding the incidence rates from each of the 5-year age groups and multiplying by 5.

**Table 2** Detection rates of the first smear and estimation of prevalence of preclinical cervical lesions in Maribo County, 1966–82

| Age[a] | Number of positive cases[b] | Number of smears | Detection rate ($\times 10^{-3}$ smears) | Estimated prevalence[c] ($\times 10^{-3}$ women) |
|---|---|---|---|---|
| 20 | 3.0 | 1382 | 2.2 | 2.7 |
| 25 | 33.3 | 4059 | 8.2 | 10.3 |
| 30 | 174.3 | 7523 | 23.2 | 29.0 |
| 35 | 141.5 | 4470 | 31.7 | 39.6 |
| 40 | 129.7 | 4006 | 32.4 | 40.5 |
| 45 | 106.7 | 3570 | 29.9 | 37.3 |
| 50 | 60.9 | 2986 | 20.4 | 25.5 |
| 55 | 3.0 | 307 | 9.8 | 12.2 |
| 60 | 1.0 | 85 | 11.4 | 14.2 |
| 60+ | 0.0 | 15 | 0.0 | 0.0 |
| 20+ | 653.4 | 28403 | 23.0 | 28.8 |

[a]The prevalence is estimated for the age *a*, by mean of [*a* – 2.5, *a* + 2.5].
[b]First smears without sufficient follow-up have been redistributed based on their cytology of the primary smear (see Table 1).
[c]Assuming an 80% sensitivity.

### *Incidence of clinical cancer during the age interval* $a_1$ *to* $a_2$ *given no screening:* $I_c$

Incidence rates for Maribo County are available from 1958 to 1962, the period just before screening started. The age-specific incidence rates for all of Denmark from 1958 to 1962 (Doll et al, 1966) did not differ significantly from those of Maribo County. The Maribo County rates show an irregular age trend because of the relatively small numbers; in the analysis we therefore used the national incidence rates. This incidence of clinical cancer is corrected for women not at risk (without a cervix uteri), using age-specific hysterectomy data from Maribo County.

### Estimation of preclinical duration

Preclinical lesions will stay prevalent for some time after which they will regress to normal or progress to clinical invasive cancer. The time they will remain screen-detectable is known as the preclinical duration. As can be seen from Figure 1C the cumulative incidence of $I_e$ up to a certain age of lesions that are no longer prevalent because of progression ($I_c$) or regression ($X$), can be estimated by subtracting the prevalence at that age from the cumulative incidence of preclinical lesions up to this age, $I_e = I_d - P$. A rough estimate of the preclinical duration is then the number of years between the age where $I_d$ reaches a certain level and the age where $I_e$ reaches the same level.

It is assumed that the duration of the preclinical stage is described by a Weibull probability distribution $F(x;m,b)$ with two parameters: mean duration $m$ and shape (or concentration parameter) $b$ (Oortmarssen and Habbema, 1991). For a given Weibull distribution the expected cumulative rate of lesions that have regressed or progressed can then be calculated for each 5-year age group $i$:

$$I^*_{ei} = \sum_{j \le i} I_{d,j} F(\bar{a}_i - \bar{a}_j)$$

where $\bar{a}_k$ is the age at midpoint of a given 5-year age group $k$, $I_{d,j}$ the incidence of preclinical lesions in the age group $j$, and $F(x)$ the Weibull distribution of the duration of the preclinical stage. The best-fitting parameters $m$ and $b$ are obtained by minimizing the

© Cancer Research Campaign 1997

**Table 3** Estimation of incidence and cumulative incidence of preclinical cervical lesions by age, based on detection rates of the third and subsequent smears, in Maribo County, 1966–82

| Age | Number of positive cases[a] | Number of women-years | Incidence of preclinical lesions (× 10⁻³ years) | Cumulative incidence of preclinical lesions (× 10⁻³ years) |
|---|---|---|---|---|
| < 20 | 0.0 | 43 | 0 | 0 |
| 20–25 | 2.6 | 1890 | 1.37 | 6.9 |
| 25–30 | 56.5 | 10698 | 5.28 | 33.3 |
| 30–35 | 51.8 | 20377 | 2.54 | 46.0 |
| 35–40 | 34.5 | 23550 | 1.47 | 53.3 |
| 40–45 | 24.7 | 19615 | 1.26 | 59.6 |
| 45–50 | 17.5 | 17752 | 0.99 | 64.5 |
| 50–55 | 13.2 | 8950 | 1.47 | 71.9 |
| 55–60 | 0.7 | 3396 | 0.21 | 72.9 |
| 60+ | 0.0 | 388 | 0 | 72.9 |
| All ages | 201.5 | 106228 | 1.90 | – |

[a]Third and subsequent smears without sufficient follow-up have been redistributed based on their cytology of the primary smear (see Table 1).

**Table 4** Incidence of clinical cervical cancer in Denmark and in Maribo County, 1958–62, and estimated incidence of clinical cervical cancer ($I_c$) for women at risk in Maribo County

| Age | Maribo County 1958–62 | | Denmark 1958–62 | | $I_c$ |
|---|---|---|---|---|---|
| | Rates ($10^{-5}$ years) | Cases | Rates ($10^{-5}$ years)[a] | Cases | Rates ($10^{-5}$ years at risk)[b] |
| < 20 | 0.0 | ( 0) | 0.1 | ( 1) | 0.1 |
| 20–24 | 0.0 | ( 0) | 2.0 | ( 15) | 2.0 |
| 25–29 | 17.3 | ( 3) | 15.9 | (112) | 16.0 |
| 30–34 | 41.1 | ( 8) | 42.1 | (306) | 42.8 |
| 35–39 | 87.3 | (19) | 75.3 | (589) | 77.8 |
| 40–44 | 65.5 | (14) | 85.1 | (651) | 90.4 |
| 45–49 | 111.9 | (24) | 85.8 | (661) | 94.5 |
| 50–54 | 98.6 | (21) | 76.7 | (568) | 86.7 |
| 55–59 | 83.3 | (16) | 69.4 | (462) | 79.5 |
| 60–64 | 55.3 | (10) | 58.6 | (345) | 67.7 |
| 65–69 | 19.4 | ( 3) | 52.6 | (252) | 61.1 |
| 70–74 | 57.5 | ( 7) | 39.0 | (145) | 45.5 |
| 75–79 | 35.7 | ( 3) | 36.8 | ( 93) | 43.1 |
| 80+ | 69.4 | ( 5) | 41.6 | ( 84) | 49.0 |

[a]Incidence of clinical cancer rates in Denmark (Doll et al, 1995).
[b]Calculated from incidence of clinical cancer in Denmark and hysterectomy rates from Maribo County.

difference between the observed ($I_e$) and the expected ($I^*_e$) cumulative incidence of lesions that have regressed or progressed.

### RESULTS

### Insufficient follow-up and predictive values

During the study period 1595 women had a positive primary smear in Maribo County: 61% of these had a histologically confirmed preclinical lesion, 31% no preclinical lesions and 9% were insufficiently followed up. For cases with sufficient follow-up, the positive predictive value of a positive smear (atypia +) for at least CIN was 67%. This value varied with the cytology of the primary smear, from 25% for 'atypical cells' to 88% for 'cells highly suspected for malignancy' (Table 1).

### Incidence and prevalence

The detection rates of preclinical lesions at the first smear are shown in Table 2. The prevalence of preclinical lesions in unscreened women, derived from these detection rates by correcting for an assumed 80% sensitivity, was 2.9% in women over 20 years of age. The highest prevalence (4%) was found at age 40.

The incidence of preclinical lesions is estimated by the detection rates per 1000 woman–years of the third and subsequent smears (Table 3), and shows a peak in age group 25–30 years. The incidence rate for women over 20 years of age was two cases per 1000 woman–years.

The incidence of clinical cancer before the start of the screening in Denmark and Maribo County increases steeply at a young age and decreases after age 50 (Table 4). The incidence of clinical cancer for women between 30 and 60 years of age was between 0.4 and 0.9 per 1000 woman–years, and the highest incidence was found for women in their forties. Incidence of clinical cancer is estimated for women at risk (with a cervix uteri) in Maribo County.

### Regression and non-progression of preclinical lesions

The estimated interval regression for women 25–50 years of age is shown in Table 5. The prevalence at age 25 was 10.3 per 1000 women (from Table 2), the cumulative incidence of preclinical lesion over the age 25 to 50 was 57.7 per 1000 woman–years (from Table 3). The prevalence at age 50 was 25.5 per 1000 women (from Table 2) and the cumulative incidence of clinical cancer of women between 25 and 50 years of age was 16.1 per 1000 woman–years (from Table 4). The estimated proportion of lesions that regressed during the interval (interval regression) was therefore $[(10.3 + 57.7) - (25.5 + 16.1)]/(10.3 + 57.7) = 0.39$ or 39%. The interval progression was $16.1/(10.3 + 57.7) = 0.24$ or 24%, and at age 50, 38% $[25.5/(10.3 + 57.7)]$ of all lesions was still prevalent.

The cumulative incidence in women aged 50–80 is 19.2 per 1000 woman–years (from Table 4). If we assume that there is no progressive onset of preclinical lesions after age 50, the proportion of prevalent preclinical lesions at age 50 that will progress to clinical cancer can be estimated to be $19.2/25.5 = 0.75$ or 75%. The minimal non-progression is then $0.39 + 0.38 \times 0.25 = 0.48$ or 48% (see Table 5). This is a minimum, as we assume no onset of preclinical lesions after age 50 and survival to age 80.

For the estimations above we used a sensitivity of 80%. Table 5 also shows calculations for a sensitivity of 70%, 80% and 90%. The impact of the different assumptions about sensitivity on the estimates is small. For a sensitivity of 70%, 80% and 90%, respectively, the estimated interval regression before age 50 years is 0.35, 0.39 and 0.42 and the non-progression rate for lesions in women aged 25–50 is, respectively, 0.47, 0.48 and 0.49.

Figure 2, for women at risk and if no screening had taken place, shows the relation between the probabilities of having developed a preclinical lesion (= incidence of preclinical lesions), of having developed a clinical cervical cancer (= incidence of clinical cancer) and of having a preclinical lesion (= prevalence). At a young age the proportion of regression and progression was small, due to the average long duration of preclinical lesions. The fact that the regression widens more than linearly with age suggests that interval regression increased with age. Between age 45 and 55 the probability of regression increases considerably.

*© Cancer Research Campaign 1997*

**Table 5** Estimation of proportion regressed, progressed and prevalent lesions for women 25–50 years of age (and confidence intervals of the estimations)

| Sensitivity (%) | $P_1$ | $I_d$ | $P_2$ | $I_c$ | Missing ($X$) | Interval regression | Interval progression | Prevalent at age 50 | Non-progression after age 25 |
|---|---|---|---|---|---|---|---|---|---|
| 80 | 10.3 | 57.7 | 25.5 | 16.1 | 26.3 | 0.39 (0.25–0.50) | 0.24 (0.20–0.27) | 0.38 (0.28–0.50) | 0.48 (0.39–0.56) |
| 70 | 11.7 | 57.7 | 29.1 | 16.1 | 24.2 | 0.35 (0.19–0.47) | 0.23 (0.20–0.27) | 0.42 (0.32–0.56) | 0.49 (0.40–0.57) |
| 90 | 9.1 | 57.7 | 22.7 | 16.1 | 28.0 | 0.42 (0.29–0.53) | 0.24 (0.21–0.28) | 0.34 (0.25–0.45) | 0.47 (0.38–0.55); |

$P_1$, prevalence at age 25 (per 1000 women); $I_d$, incidence preclinical lesions (per 1000 woman–years) between ages 25 and 50; $P_2$, prevalence at age 50 (per 1000 women); $I_c$, incidence clinical cancer (per 1000 women) between age 25 and 50. Missing ($X$), $(P_1 + I_d) - (P_2 + I_c)$; interval regression, $[(P_1 + I_d) - (P_2 + I_c)]/(P_1 + I_d)$; interval progression, $I_c/(P_1 + I_d)$; prevalent lesions at age 50, $P_2/(P_1 + I_d)$. Non-progression after age 25 = interval regression + prevalent at age 50 × non-progression after age 50 (= $I_c$, women 50–80, $/P_2$).

**Table 6** Estimated mean duration *m* and shape *b* of the Weibull distribution function of the preclinical duration (and confidence interval)

| Sensitivity (%) | Mean duration *m* (years) | Shape *b* |
|---|---|---|
| 70 | 17.6 (14.8–23.8) | 5.8 (2.0–∞) |
| 80 | 15.7 (13.4–24.6) | 3.2 (1.2–∞) |
| 90 | 14.2 (10.0–181.9) | 2.0 (0.3–∞) |

**Figure 2** The probability of developing a preclinical cervical lesion, and probabilities of prevalence, progression and regression, under the assumption that there is no onset of preclinical lesions in women over 50 years of age. The preclinical incidence is the prevalence (from Table 2) at age 25 + the cumulative incidence of preclinical cervical lesions (from Table 3) in women after age 25. The cumulative incidence of preclinical cervical lesions ($I_d$ fromTable 3) is converted into probabilities using the formula: $P = 1 - \exp(-\Sigma I_d)$. The clinical incidence in this figure is the cumulative incidence of clinical cervical cancer ($I_c$ from Table 4).

In the estimation of non-progression we assumed that all women survive up to age 80. However, a proportion of women with progressive lesions will die from other causes before the cancer is diagnosed clinically. After correction for mortality [using 1993 mortality rates (Danmarks Statistik, 1993)], the cumulative incidence of clinical cancer for women aged 50–80 years will be 17.0. The proportion of lesions which progress after age 50 will be 17.0/25.5 = 0.66 or 66%. Under these assumptions, the minimal non-progression is 0.39 + 0.38 × 0.34 = 0.51 or 51%.

### Duration of the preclinical lesion

For a sensitivity of 70%, 80% and 90%, respectively, we estimated the mean duration of the preclinical lesion to be 17.6, 15.7 and 14.2 years, respectively (Table 6). The estimated duration is only marginally influenced by the sensitivity.

### DISCUSSION

The natural history of the detectable preclinical phase of cervical cancer can only be studied indirectly on the basis of screening data. We estimated non-progression and duration from the Maribo County data using a two-step procedure. Firstly, the prevalence and incidence rates of preclinical disease were estimated from the observed detection rates. Secondly, the non-progression and duration were assessed, also using the prescreening incidence of clinical cancer as a proxy for the expected incidence if no screening had taken place. The main findings were that at least 48% of the lesions in women between 25 and 50 years of age do not progress into clinical cancer. If one accounts for death from causes other than cervical cancer, this minimum percentage increases to around 51%. The mean duration of all preclinical lesions was estimated at 16 years.

Our estimate for non-progression is based on detected lesions. Short regressive lesions would have stayed undetected if they developed and regressed within a screening interval. This causes an underestimation of the proportion of non-progression. The side-effects associated with the detection of non-progressive lesions however, are not underestimated.

The estimation procedure for regression and non-progression was performed under the assumption that there is no cohort effect in the observed period. In an age–period–cohort analysis of incidence of clinical cancer in Denmark before 1967, we found that

 © Cancer Research Campaign 1997

women born in the years 1918–29 were presumably at higher risk than women born later. If such a cohort effect occurred, the prevalence at age 50 and the incidence of clinical cancer were overestimated, leading to an underestimation of the non-progressive rate. Such a cohort effect would also lead to a decrease in detection rates with ascending calendar years. However we found that the detection rates for 1975–82 were in fact higher than the rates of the period 1966–74. This cannot be explained by an increase in incidence of cervical cancer at a young age, because such an increase was only modest in Denmark and seen only after 1983. Furthermore, the increase in detection rates was seen in all age groups. An explanation for this observation could be a drift over time towards a lower 'follow-up threshold' and in consequence, a higher sensitivity at the expense of specificity.

We used the incidence of invasive cervical cancer before the screening programme started, to estimate the incidence of clinical cancer in the screened women if no screening had taken place. For participants however, the incidence of cervical cancer has been found to be relatively low (Berget, 1979; Magnus et al 1987; Oortmarssen and Habbema, 1991). Not accounting for this lower incidence leads to an underestimation of the non-progression. Assuming an incidence level in participants of 74% of the total population (Oortmarssen and Habbema, 1991), the minimal non-progression fraction would increase from 48% to 54%, or from 51% to 58% if one accounts for death from other causes.

Using prevalence and incidence of preclinical lesions and incidence of clinical cancer from British Columbia, Canada in 1949–69 (Boyes et al, 1982), we estimated that 48% of the lesions in women between 25 and 50 years of age regressed before the age of 50, which is somewhat higher than the 39% found in Maribo County. Gustafsson et al (1989) analysed Swedish screening data and estimated the progression rate for carcinoma *in situ* at 12%, which is considerably lower than the maximum proportion of progression for all preclinical lesions of 52% found for Maribo County. At least part of the difference is explained by the fact that the Swedish study included onset of preclinical lesions also after the age of 50. Hence, a smaller proportion of the clinical cancer after age 50 is explained by the preclinical cancer developed before this age.

The estimated mean duration of the preclinical lesions in Maribo County was 16 years, compared with 15.8 years in British Columbia (Oortmarssen and Habbema, 1991) and 17.3 years in Sweden (Gustafsson and Adami, 1989). These estimates are remarkably similar, despite differences in calculation methods and between the screening programmes. These estimates of duration represent an average for the regressive, stable and progressive lesions. They may well have different mean durations, but it is not possible to separate these using this rather straightforward analysis. For the purpose of screening it is the duration of progressive lesions that is important. Van Oortmarssen et al (1995) showed that an average duration of 15.8 years for preclinical progressive disease is compatible with the interval cancer data collected by the IARC in the eighties, which also involved data derived from the Maribo County data set studied in this paper (IARC, 1986; Lynge and Poll, 1986*b*).

Our study confirmed that non-progression is a common phenomenon that should be taken into account in the evaluation of cervical cancer screening. Of the screened women in Maribo County, 1.5% had a positive smear with at least atypia, and the majority of these women were followed up: one-third with a negative diagnosis and two-thirds with a histologically confirmed preclinical lesion. Our analysis shows that at least half of these confirmed preclinical lesions would not have progressed into clinical cancer in the women's lifetime. Thus, among the women screened in Maribo County, 5 per 1000 were diagnosed with a false-positive smear, 5 per 1000 were diagnosed and treated for a non-progressive preclinical lesion, and 5 per 1000 were diagnosed and treated for a preclinical lesion that would otherwise have developed into invasive cervical cancer. The screened women thus pay a price in overtreatment in order to minimize the incidence and mortality from cervical cancer. But given the severity of the disease and the relatively mild treatment with conization, cryotherapy and laser therapy, this price – as it is estimated for Maribo County in the period studied – seems reasonable.

Analysis of data from the cervical cancer screening programme in Bristol (Raffle et al, 1995) in the years 1988–93, showed that 7% of the screened women had smear abnormalities, and 2.7% were referred to colposcopy. This latter proportion is close to double that for Maribo County, which is high, taking into account that incidence of invasive cancer was, and still is, appreciably higher in Denmark than in the UK (37 per 100 000 in Denmark in 1958–62 (Doll et al, 1966) and 17 per 100 000 in England and Wales in 1960–62 (Doll et al, 1970); 16 per 100 000 in Denmark and 12 per 100 000 in England and Wales in 1983–87 (Parkin et al, 1992). The cervical cancer screening data from the Netherlands from 1987 to 1990 show more than 10% positive smears (PALGA, 1992). Similarly, up to 10% of cervical smears from the United States currently have ASCUS or more severe abnormalities (Singer, 1995) and 5% of all smears have low-grade squamous intraepithelial lesions (Kurman et al, 1994).

It is clear that there is a considerable variation in the cost in terms of overtreatment paid by different populations to prevent progressive preclinical lesions. It is therefore worrying that over recent decades, there has been a tendency in several countries to advise more intensive follow-up after slightly abnormal Pap smears. Owing to the estimated long duration in combination with a relatively high sensitivity for the preclinical lesion, the extra incidence and mortality reduction from more intense follow-up of slightly abnormal Pap smears in regular attenders, to a 3–5 yearly screening, is expected to be very low.

### ABBREVIATIONS

CIN, cervical intraepithelial neoplasia; ASCUS, atypical squamous cells of undetermined significance.

### ACKNOWLEDGEMENT

This study was financially supported by the Danish Cancer Society.

### REFERENCES

Berget A (1979) Influence of population screening on morbidity and mortality of cancer of the uterine cervix in Maribo Amt. *Dan Med Bull* **26**: 91–100

Boyes DA, Morrison B, Knox EG, Draper FJ and Miller AB (1982) A cohort study of cervical cancer screening in British Columbia. *Clin Invest Med* **5**: 1–29

Danmarks Statistik (1995) *Vital Statistics 1993*. Danmarks Statistik: Copenhagen (in Danish)

Doll R, Payne P and Waterhouse J (eds) (1966) *Cancer Incidence in Five Continents. A Technical Report*. Springer: Berlin.

Doll R, Muir C and Waterhouse J (eds) (1970) *Cancer incidence in Five Continents, Vol II*. Springer: Berlin

© Cancer Research Campaign 1997

Gustafsson L and Adami HO (1989). Natural history of cervical neoplasia: consistent results obtained by an identification technique. *Br J Cancer* **60:** 132–141

IARC Working Group on Evaluation of Cervical Cancer Screening Programmes (1986) Screening for squamous cervical cancer: duration of low risk after negative results of cervical cytology and its implication for screening programmes. *Br Med J* **293:** 659–664

Kleinbaum DG, Kupper LL and Mogenstern H (1982). *Epidemiologic Research, Principles and Quantitative Methods.* pp. 296–300. Lifetime Learning Publications: London

Kurman RJ, Henson DE, Herbst AL, Noller KL and Schiffman MH for The 1992 National Cancer Institute Workshop (1994) Interim guidelines for management of abnormal cervical cytology. *JAMA* **271:** 1866–1869.

Lynge E and Poll P (1986*a*) Risk of cervical cancer following negative smears in Maribo County, Denmark, 1966–1982. In *Screening of Cancer of the Uterine Cervix,* Miller AB, Day N, Hakama M (eds) pp. 69–86. International Agency for Research on Cancer: Lyon

Lynge E and Poll P (1986*b*) Incidence of cervical cancer following negative smear. A cohort study from Maribo County, Denmark. *Am J Epidemiol* **124:** 345–352

Magnus K, Langmark F and Andersen A (1987) Mass screening for cervical cancer in Ostfold county in Norway 1959–77. *Int J Cancer* **39:** 311–316

Oortmarssen GJ Van and Habbema JDF (1991) Epidemiological evidence for age-dependent regression of pre-invasive cervical cancer. *Br J Cancer* **64:** 559–565

Oortmarssen GJ Van and Habbema JDF (1995) Duration of preclinical cervical cancer and reduction in incidence of invasive cancer following negative pap smears. *Int J Epidemiol* **24:** 300–307

Ostor AG (1993) Natural history of cervical intraepethelial neoplasias; a critical review. *Int J Gynecol Pathol* **12:** 186–192

Singer A (1995) Cervical cancer screening: state of the art. *Bailliere's Clin Obstet Gynaecol* **9(1):** 39–64

Parkin DM, Muir CS, Whelan SL, Gao Y-T, Ferlay J and Powell J (eds) (1992) *Cancer Incidence in Five Continents, Vol VI.* IARC Scientific Publications: Lyon

Pathological National Automated Archive (PALGA) (1992) *Results of Retrieval Action on Cervical Cytology from 1987–1990.* PALGA: Amsterdam

Raffle AE, Alden B and Mackenzie EFD (1995) Detection rates for abnormal cervical smears: what are we screening for? *Lancet* **345:** 1469–1473

Wilson JMG and Jungner F (1968) *Principles and Practice of Screening for Disease.* Public Health Papers 34. World Health Organization: Geneva

*© Cancer Research Campaign 1997*